# Supplementary material for: N-Terminal Pro-B-Type Natriuretic Peptide (NT-proBNP)—A Prognostic Biomarker in Older and/or Frail Adults with Advanced Gastroesophageal Cancer: A Post Hoc Analysis of the GO2 Clinical Trial
Source: Cancers (Basel). 2025 Feb 10;17(4):601. doi: 10.3390/cancers17040601 (PMC11852610; doi:10.3390/cancers17040601)
Supplement: Supplementary file 1 [file cancers-17-00601-s001.zip › cancers-3368079-supplementary.pdf]

Supplementary Data

Table S1. Demographics of included patients compared to whole GO2 patient population.

	BNP study (N=241)	GO2 trial (N=558)
**Age (years)**		
Mean (SD)	74.9 (7.20)	75.2 (6.94)
Median [Min, Max]	76.0 [52.0, 89.0]	77.0 [51.0, 96.0]
**Age Group**		
<75	104 (43.2%)	227 (40.7%)
≥75	137 (56.8%)	331 (59.3%)
**Sex**		
Male	187 (77.6%)	412 (73.8%)
Female	54 (22.4%)	146 (26.2%)
**Site of primary**		
Oesophagus	100 (41.5%)	219 (39.2%)
GOJ	59 (24.5%)	131 (23.5%)
Gastric	81 (33.6%)	206 (36.9%)
Missing	0 (0%)	2 (0.4%)
**ECOG PS**		
0	33 (13.7%)	72 (12.9%)
1	128 (53.1%)	293 (52.5%)
2+	80 (33.2%)	191 (34.2%)
Missing	0 (0%)	2 (0.4%)
**Metastases**		
Present	168 (69.7%)	368 (65.9%)
Absent	73 (30.3%)	190 (34.1%)
**Albumin (g/dl)**		
Mean (SD)	34.7 (13.6)	34.9 (13.6)
Median [Min, Max]	36.0 [2.0, 49.0]	36.0 [2.0, 49.0]
Missing	0 (0%)	2 (0.4%)
**Hb**		
Mean (SD)	12.8 (7.4)	13.3 (9.73)
Median [Min, Max]	12.1 [6.60, 18.0]	12.2 [5.60, 18.0]
Missing	1 (0.4%)	2 (0.4%)
**Dose Level**		
100% OX	77 (32.0%)	170 (30.5%)

	BNP study (N=241)	GO2 trial (N=558)
80% OX	83 (34.4%)	171 (30.6%)
60% OX	81 (33.6%)	195 (34.9%)
BSC	0 (0%)	22 (3.9%)
**GO2 Frailty Group**		
No frailty	49 (20.3%)	96 (17.2%)
Mildly frailty	58 (24.1%)	132 (23.7%)
Severely frailty	134 (55.6%)	328 (58.8%)
Missing	0 (0%)	2 (0.4%)

Table S2. ProBNP levels with upper limit of normal at individual centre.

Patient	ProBNP	BNP Lower Limit of Normal	BNP Upper Limit of Normal
1	582	0	300
2	1	400	2000
3	298	0	250
4	47	0	100
5	592	400	1999
6	220	0	399
7	477	400	2000
8	893	400	1999
9	136	0	400
10	646	0	399
11	301	0	400
12	610	0	249
13	232	0	49
14	1072	400	1999
15	514	0	400
16	405	0	249
17	3532	400	2000
18	266	0	100
19	25	0	160
20	16	0	400
21	1255	0	250
22	1998	0	400
23	128	400	2000
24	574	0	100
25	161	400	2000
26	314	0	100
27	103	0	249
28	789	0	250
29	1022	0	400
30	178	0	400
31	127	0	161
32	678	400	2000
33	683	400	2000
34	28	0	47
35	306	306	306
36	433	0	249
37	298	0	400
38	425	400	2000
39	220	0	161
40	19	0	400
41	213	400	2000
42	538	250	250

43	133	0	300
44	174	0	400
45	164	0	400
46	11	0	47
47	397	0	400
48	116	0	100
49	190	0	75
50	138	0	200
51	497	0	400
52	274	0	400
53	197	0	400
54	181	0	400
55	362	0	249
56	328	0	400
57	386	0	300
58	59	0	400
59	2897	0	400
60	147	0	400
61	544	0	400
62	123	0	400
63	265	0	400
64	583	400	2000
65	413	400	2000
66	1917	0	249
67	362	0	400
68	122	400	2000
69	501	0	400
70	420	0	1000
71	283	0	400
72	164	0	400
73	539	0	400
74	2322	0	250
75	230	0	400
76	1210	400	2000
77	118	0	738
78	83	0	99
79	803	0	400
80	338	400	2000
81	50	0	100
82	312	0	99
83	604	0	100
84	745	0	250
85	443	0	300
86	499	400	2000
87	2101	400	2000
88	1626	0	300

89	453	249	249
90	165	0	400
91	98	400	2000
92	181	400	2000
93	100	0	300
94	132	0	260
95	126	0	400
96	220	0	300
97	662	400	2000
98	5681	400	2000
99	1395	400	2000
100	183	400	2000
101	106	0	400
102	460	0	400
103	33	0	1000
104	282	0	400
105	373	0	400
106	172	0	250
107	123	400	2000
108	57	400	2000
109	254	0	100
110	337	0	400
111	325	0	400
112	148	0	100
113	101	0	400
114	123	0	99
115	477	0	300
116	426	0	260
117	767	0	400
118	1127	0	250
119	1312	400	2000
120	124	0	400
121	550	0	241
122	142	0	486
123	170	400	2000
124	195	0	400
125	497	0	300
126	120	400	2000
127	200	0	400
128	202	0	250
129	100	0	400
130	286	0	400
131	223	400	2000
132	100	0	250
133	330	0	161
134	1459	400	2000

135	201	0	150
136	203	0	400
137	194	0	99
138	214	400	2000
139	241	0	400
140	67	0	100
141	179	0	400
142	232	0	249
143	147	0	400
144	294	0	400
145	1275	399	399
146	1185	0	400
147	101	0	400
148	55	0	100
149	99	400	2000
150	630	0	400
151	2580	0	400
152	1316	249	249
153	303	0	400
154	253	0	250
155	1036	400	2000
156	148	400	400
157	252	400	2000
158	175	0	400
159	180	400	2000
160	35	0	40
161	74	100	400
162	1690	0	2000
163	917	0	399
164	160	0	300
165	680	0	300
166	766	0	399
167	374	400	2000
168	93	0	400
169	38	0	300
170	443	400	2000
171	302	0	399
172	225	0	400
173	248	0	400
174	203	0	99
175	141	0	400
176	683	0	400
177	295	0	400
178	293	0	400
179	291	0	400
180	905	0	400

181	65	0	400
182	190	0	400
183	376	0	400
184	166	0	400
185	1247	0	400
186	71	0	400
187	300	0	400
188	887	399	2000
189	223	0	400
190	567	0	400
191	131	0	300
192	78	0	400
193	140	0	400
194	46	0	400
195	186	0	400
196	4127	0	400
197	862	0	400
198	82	0	250
199	80	0	399
200	342	0	300
201	165	0	400
202	484	0	400
203	814	0	400
204	292	1	486
205	521	400	2000
206	1321	401	1999
207	275	0	399
208	665	0	250
209	498	0	400
210	304	0	249
211	278	0	400
212	100	1	486
213	43	0	99
214	389	250	400
215	104	0	399
216	1310	401	1999
217	1304	0	400
218	208	0	399
219	422	0	300
220	233	0	400
221	392	0	400
222	246	401	1999
223	237	0	300
224	82	0	300
225	86	0	400
226	38	0	300

227	349	0	400
228	480	0	400
229	807	0	400
230	459	0	400
231	2750	0	399
232	386	15	400
233	190	0	400
234	417	1	486
235	311	0	400
236	109	1	486
237	364	0	300
238	412	0	300
239	208	0	300
240	339	0	400
241	414	0	400
242	1103	401	1999
243	132	0	300
244	200	0	300
245	44	0	399
246	393	0	400
247	1068	0	300
248	276	0	400
249	627	0	400
250	259	75	400
